# Inclination towards research and the pursuit of a research career among medical students: an international cohort study

**DOI:** 10.1186/s12909-018-1202-6

**Published:** 2018-05-02

**Authors:** Tam Cam Ha, Sheryl Ng, Cynthia Chen, Sook Kwin Yong, Gerald C. H. Koh, Say Beng Tan, Rahul Malhotra, Fernando Altermatt, Arnfinn Seim, Aya Biderman, Torres Woolley, Truls Østbye

**Affiliations:** 1 Duke-NUS Medical School, National University of Singapore, Singapore, Singapore; 2 The University of Wollongong, Wollongong, Australia; 3 Saw Swee Hock School of Public Health, National University of Singapore, Singapore, Singapore; 4 Pontificia Universidad Catolica de Chile, Santiago, Chile; 5 Norwegian University of Science and Technology (NTNU), Trondheim, Norway; 6 Ben-Gurion University, Beer-Sheva, Israel; 7 James Cook University, Townsville, Australia; 8 Duke University Medical Centre, Durham, NC USA

**Keywords:** Inclination, Medical students, Research career, Research, Cohort

## Abstract

**Background:**

Involvement of clinicians in biomedical research is imperative for the future of healthcare. Several factors influence clinicians’ inclination towards research: the medical school experience, exposure to research article reading and writing, and knowledge of research. This cohort study follows up medical students at time of graduation to explore changes in their inclination towards research and pursuing a research career compared to their inclination at time of entry into medical school.

**Methods:**

Students from medical schools in six different countries were enrolled in their first year of school and followed-up upon graduation in their final year. Students answered the same self-administered questionnaire at both time points. Changes in inclination towards research and pursuing a research career were assessed. Factors correlated with these changes were analysed.

**Results:**

Of the 777 medical students who responded to the study questionnaire at entry into medical school, 332 (42.7%) completed the follow-up survey. Among these 332 students, there was no significant increase in inclination towards research or pursuing a research career over the course of their medical schooling. Students from a United States based school, in contrast to those from schools other countries, were more likely to report having research role models to guide them (51.5% vs. 0%–26.4%) and to have published in a peer-reviewed journal (75.7% vs. 8.9%–45%). Absence of a role model was significantly associated with a decrease in inclination towards research, while an increased desire to learn more about statistics was significantly associated with an increase in inclination towards pursuing a research career.

**Conclusion:**

Most medical students did not experience changes in their inclination towards research or pursuing a research career over the course of their medical schooling. Factors that increased their inclination to undertaking research or pursuing a research career were availability of a good role model, and a good knowledge of both the research process and the analytical tools required.

## Background

Biomedical research has the potential to generate positive health and economic outcomes [1]. However, studies across many countries have reported a decreasing trend of physicians undertaking clinical research [2]. Since 2000, the number of physician-scientists has not kept pace with the overall growth of the medical research community [3]. The progress of biomedical research is highly dependent on physicians being actively engaged in research. Physicians are more able to pursue clinically relevant research as they are in regular contact with patients, have a deeper understanding and appreciation of diseases and the clinical environment.

In view of the decreasing number of physician-scientists, various research councils and institutes across different countries are increasing their focus on training ‘clinician scientists’ (medical graduates who primarily pursue a career in biomedical research) and ‘clinician investigators’ (medical graduates who primarily are involved in patient care, but are also involved in research) [4].

Individual student experiences in medical school are the most important determinant in clinicians choosing to pursue a career in research and cultivating a research interest [5–7]. For example, several key factors associated with a career in research include participation in research projects or electives and authoring research papers while in medical school [8], involvement in research training and experience while in medical school [9], and the desire to pursue a graduate research degree along with a medical degree [10]. However, a recent study showed only a minority of first-year medical students across different countries and different medical schools were inclined towards a career in research [4].

This article reports on medical students’ inclination towards a research career at the time of graduation, involving the same cohort as Tan et al. [4]. In that original study, medical students were assessed in terms of their confidence with biomedical research methodology, ability to interpret and understand biomedical literature, and their inclination towards research at the point of entry into medical school. The objective of this follow-up study is to determine whether medical students from different medical schools worldwide experienced any changes in their inclination towards research and the pursuit of a research career from first-year to the time of graduation, and which factors may have encouraged or discouraged such changes in inclinations.

## Methods

This prospective cohort study is the follow-up to a previously published baseline survey [4]. Medical schools from eleven universities in ten countries across five continents collaborated on this comparison study. The study design, methodology and study implementation have been previously documented [4]. In brief, a self-administered, non- anonymised questionnaire was used for a baseline study of first year medical students. The identical questionnaire was used for the prospective follow-up in students’ final year. The inclination of students towards research and a biomedical research career were assessed by comparing the questions at the two time points (first year medical school vs final year medical school). The pilot-tested questionnaire was developed by the study team after extensive, iterative discussions in person, via telephone and email, and then later translated from English into three languages (Portuguese, Spanish and Hebrew). Questions included exposure to medical research, experience in performing medical research, and perceived research capabilities.

### Follow-up

The medical students who responded to the baseline questionnaires were re-contacted near time of graduation. The questionnaire was identical as used in the baseline survey but with minor modifications, as each site required minor variations in procedures for administering the questionnaires due to differences in duration and course structure for each medical school. The minor modifications of the questionnaire were paraphrasing the questions in the baseline questionnaire with suitable tense used so as to be more grammatically appropriate to the final year medical students. The site investigators administered the questionnaire to final-year, graduating students during one of their compulsory classes, in person. The students then completed the questionnaires individually. The hard copies of the completed questionnaires were sent to the study team in Singapore for data analysis. Study participation was voluntary and informed written consent obtained from each student. Ethics approval was obtained from the NUS Institutional Review Board (NUS-IRB Ref No 08–274). Each site also obtained the necessary Institutional Review Board/Ethics Committee approval as required by the institution and country’s regulations.

### Statistical analysis

Descriptive summary statistics were computed for each university, and the data combined for all universities. Demographic data were taken from the baseline study while the curriculum data were taken from the follow-up survey. Chi-square tests were used to test bivariate associations between institutions and respondent characteristics. The categories relating to understanding and capabilities in research were biostatistics, research ethics and study design. Changes in inclination towards research and biomedical career were defined as changes in inclination from beginning to end of medical school (first and final year). Respondents who went from a ‘No’ to ‘Yes’/ ‘Undecided’ and ‘Undecided’ to ‘Yes’ were deemed to have experienced an increase in inclination, while respondents who went from a ‘Yes’ to ‘No’/ ‘Undecided’ and ‘Undecided’ to ‘No’ were deemed to have experienced a decrease in inclination.

Multinomial logistic regression was used to identify factors associated with two outcomes of interest; increase or decrease in inclination towards research, and increase or decrease inclination towards a research career (with no change in inclination as the reference comparison). A stepwise approach was used to remove any predictors which were not significant and odds ratios (OR) with confidence intervals reported. A sensitivity analysis was carried out for robustness of results: missing data of non-respondents in the follow-up survey was entered with their baseline information and full analyses were again performed. All statistical analyses were performed using R software (R. RStudio, Inc., Boston, MA, USA 2015). Significance was set at *p* < 0.05 for all analyses.

## Results

Among the 11 universities in 10 countries that were part of the original study, 7 universities from 6 countries responded to the follow-up. These universities were: James Cook University (JCU) Australia; Pontificia Universidad Catolica de Chile (PUC) Chile; Ben-Gurion University (BGU) Israel; Duke University USA; Norwegian University of Science and Technology (NTNU) Norway; and the National University of Singapore (NUS) (students from both Yong Loo Lin School of Medicine and Duke-NUS Medical School) Singapore (Table 1). Singapore data were presented as a combination of the two schools because the sample size for Duke-Nus Medical School was too small to be presented as a stand-alone site.

**Table 1:** Demographic profiles, research inclination and previous research experience of the medical students from various medical schools

		Institutions^a^					
	Total	JCU	PUC	BGU	DUMC	NTNU	NUS
First year	777	167	98	41	82	108	281
Final year	332	45	20	29	37	47	154
N (%)							
Age at first year							
Median	19	17	18	24	24	22	19
(Min, Max)	(16, 33)	(16, 22)	(17, 23)	(19, 27)	(22, 33)	(19, 25)	(18, 28)
Gender							
Female	179 (53.9)	25 (55.6)	9 (45.0)	12 (41.4)	15 (40.5)	35 (74.5)	83 (53.9)
Male	153 (46.1)	20 (44.4)	11 (55.0)	17 (58.6)	22 (59.5)	12 (25.5)	71 (46.1)
Highest Academic Qualification							
No previous degree	239/327 (73.1)	40/44 (90.9)	19 (95.0)	25/28 (89.3)	0 (0.0)	35/45 (77.8)	120/153 (78.4)
Diploma	18/327 (5.5)	3/44 (6.8)	1 (5.0)	1/28 (3.6)	0 (0.0)	3/45 (6.7)	11/153 (7.2)
Bachelors	61/327 (18.7)	0 (0.0)	0 (0.0)	2/28 (7.1)	32 (86.5)	7/45 (15.6)	19/153 (12.4)
Masters	7/327 (2.1)	1/44 (2.3)	0 (0.0)	0 (0.0)	4 (10.8)	0 (0.0)	2/153 (1.3)
PhD & above	2/327 (0.6)	0 (0.0)	0 (0.0)	0 (0.0)	1 (2.7)	0 (0.0)	1/153 (0.7)
Inclination towards research^b^							
No change	133/323 (41.2)	17 (37.8)	7 (35.0)	11/28 (39.3)	17/35 (48.6)	25 (53.2)	56/148 (37.8)
Decrease	90/323 (27.9)	8 (17.8)	5 (25.0)	7/28 (25.0)	6/35 (17.1)	14 (29.8)	50/148 (33.8)
Increase	100/323 (31.0)	20 (44.4)	8 (40.0)	10/28 (35.7)	12/35 (34.3)	8 (17.0)	42/148 (28.4)
Inclination towards a biomedical career^b^							
No change	137/329 (41.6)	17/44 (38.6)	3 (15.0)	11/28 (39.3)	14/36 (38.9)	25 (53.2)	67 (43.5)
Decrease	118/329 (35.9)	18/44 (40.9)	15 (75.0)	6/28 (21.4)	8/36 (22.2)	10 (21.3)	61 (39.6)
Increase	74/329 (22.5)	9/44 (20.5)	2 (10.0)	11/28 (39.3)	14/36 (38.9)	12 (25.5)	26 (16.9)
Done research during medical school							
No	41/326 (12.6)	22 (48.9)	2 (10.0)	1 (3.4)	0 (0.0)	0 (0.0)	17/152 (11.2)
Yes	285/326 (87.4)	23 (51.1)	18 (90.0)	28 (96.6)	37 (100.0)	47 (100.0)	135/152 (88.8)
Taken a course in research during medical school outside regular curriculum							
No	255 (76.8)	41 (91.1)	18 (90.0)	17 (58.6)	20 (54.1)	41 (87.2)	118 (76.6)
Yes	77 (23.2)	4 (8.9)	2 (10.0)	12 (41.4)	17 (45.9)	6 (12.8)	36 (23.4)
Taken a course in research ethics during medical school outside regular curriculum							
No	295/326 (90.2)	42/44 (95.4)	20 (100.0)	23/28 (82.1)	25 (67.6)	47 (100.0)	138/151 (91.4)
Yes	32/326 (9.8)	2/44 (4.6)	0 (0.0)	5/28 (17.9)	12 (32.4)	0 (0.0)	13/151 (8.6)
Had articles accepted or published in peer-reviewed journals while in medical school							
No	218 (65.7)	41 (91.1)	11 (55.0)	25 (86.2)	9 (24.3)	36 (76.6)	96 (62.3)
Yes	114 (34.3)	4 (8.9)	9 (45.0)	4 (13.8)	28 (75.7)	11 (23.4)	58 (37.7)
Had an experience during medical school that put off respondent from research							
No	248/326 (74.9)	36 (80.0)	6 (30.0)	28 (96.6)	22 (59.5)	42/46 (91.3)	114 (74.0)
Yes	83/326 (25.1)	9 (20.0)	14 (70.0)	1 (3.4)	15 (40.5)	4/46 (8.7)	40 (26.0)
Had an experience during medical school that ignited interest in research							
No	230/326 (70.3)	26/43 (60.5)	9 (45.0)	22 (75.9)	18 (48.6)	35 (74.5)	120/151 (79.5)
Yes	97/326 (29.7)	17/43 (39.5)	11 (55.0)	7 (24.1)	19 (51.4)	12 (25.5)	31/151 (20.5)
Read biomedical journals at least once every 6 months							
No	163 (49.1)	15 (33.3)	10 (50.0)	14 (48.3)	13 (35.1)	17 (36.2)	94 (61.0)
Yes	169 (50.9)	30 (66.7)	10 (50.0)	15 (51.7)	24 (64.9)	30 (63.8)	60 (39.0)
Presence of role model during medical school							
No	232/326 (73.7)	31/40 (77.5)	15/15 (100.0)	21/28 (75.0)	18 (48.6)	38 (80.9)	109/148 (73.6)
Yes	83/326 (26.3)	9/40 (22.5)	0/15 (0.0)	7/28 (25.0)	19 (51.4)	9 (19.1)	39/148 (26.4)
Percentage of work time envisioned to spend on research							
Mean (SD)	11.6 (12.7)	9.1 (11.9)	7.7 (4.2)	10.7 (4.32)	23.0 (22.0)	11.3 (13.2)	10.2 (9.8)

^a^*JCU* James Cook University, *PUC*, Pontificia Universidad Catolica de Chile, *BGU* Ben-Gurion University, *DUMC* Duke University Medical Centre, *NTNU* Norwegian University of Science and Technology, *NUS* National University of Singapore (inclusive of Yong Loo Lin School of Medicine and from Duke-NUS Graduate Medical School)

^b^Increase: No to Undecided or Yes, or Undecided to Yes; Decrease: Yes to Undecided or No; or Undecided to No

A total of 777 responses were provided in the baseline survey; of these, 332 (42.7%) responded to the final year survey. The demographics of the students who responded to the follow-up study are described in Table 1. The median age at baseline was 19 years (range; 16–33 years), with BGU and Duke University having the highest median age of 24 years, and JCU Australia the lowest at 17 years. Duke University (USA) and Duke-NUS (Singapore) were the only institutions with solely graduate students, so most students (73.1%) overall did not have a previous degree. There was no significant difference in baseline characteristics between the respondents and non-respondents of this follow-up survey, but most of the non-respondents did not have any prior experience with research (data not shown).

Most students experienced no change in inclination towards research except JCU (44.4% vs. 37.8%) and PUC (40.0% vs. 35.0%), where more students increased their inclination towards research compared to those who had no change in inclination towards research. Also, most students reported no change in their inclination towards research careers.

The majority of students had performed some research during the course of their medical school but had not taken any additional courses in research or ethics outside their regular curriculum; this was observed across all the institutions. While few students overall had published an article in peer-reviewed journals when in medical school, Duke University students reported otherwise with 75.7% having done so.

Most students indicated that they did not have any role model to guide their research during the course of the medical programme except Duke University, where more than half (51.4% vs. 0%–26.4%) of the students reported in the affirmative. In addition, Duke University students envisioned having the highest percentage of their postgraduate work time spent undertaking research (mean: 23% vs. 7.7%–11.6%).

Table 2 presents the change in the self-reported capabilities and confidence of the medical students. While no significant change in students’ understanding or confidence towards research methods was observed, BGU had the highest proportion of increased understanding in biostatistics, capabilities in study design and data analysis, while NTNU and NUS had high proportions of students with increased understanding of research ethics, and Duke University recorded the highest proportion of students with increased confidence in publication ethics. The aspects which various medical schools’ students showed consistent trend in increasing confidence were understanding ethics terms found in research journals and evaluating diagnostic tests.

**Table 2:** Proportion of medical students who changed in agreement to different statements and confidence on various aspects biomedical research, n (%)

			Institutions^a^						
		Total	JCU	PUC	BGU	DUMC	NTNU	NUS	
		(*n* = 332)	(*n* = 45)	(*n* = 20)	(*n* = 29)	(*n* = 37)	(*n* = 47)	(*n* = 154)	
Agreement with statement:	N^c^ (%)								*P*-value^b^
*Biostatistics*									
I would like to learn more about biostatistics.	Decreased	37/329 (11.2)	4/44 (9.1)	0 (0.0)	4 (13.8)	2/36 (5.6)	9 (19.1)	18/153 (11.8)	< 0.001*
	Increased	62/329 (18.8)	3/44 (6.8)	11 (55.0)	11 (37.9)	4/36 (11.1)	10 (21.3)	23/153 (15.0)	
I can understand almost all of the statistical terms that I encounter in journal articles.	Decreased	43/330 (13.0)	8 (17.8)	5 (25.0)	1 (3.4)	3 (8.1)	2 (4.3)	24/152 (15.8)	0.016*
	Increased	111/330 (33.6)	14 (31.1)	9 (45.0)	16 (55.2)	8 (21.6)	15 (31.9)	49/152 (32.2)	
It is easy to manipulate statistics to support results desired by investigators.	Decreased	43/328 (13.1)	11 (24.4)	4 (20.0)	1/28 (3.6)	1/36 (2.8)	1 (2.1)	25/152 (16.4)	< 0.001*
	Increased	62/328 (18.9)	5 (11.1)	2 (10.0)	7/28 (25.0)	4/36 (11.1)	15 (31.9)	29/152 (19.1)	
To be an intelligent reader of the biomedical literature, it is necessary to know something about statistics.	Decreased	4/330 (1.2)	1/44 (2.3)	0 (0.0)	0 (0.0)	0 (0.0)	0 (0.0)	3/153 (2.0)	< 0.001*
	Increased	9/330 (2.7)	6/44 (13.6)	0 (0.0)	0 (0.0)	0 (0.0)	0 (0.0)	3/153 (2.0)	
*Research Ethics*									
I would like to learn more about research ethics.	Decreased	77/327 (23.5)	8 (17.8)	6/19 (31.6)	7 (24.1)	9 (24.3)	11 (23.4)	36/150 (24.0)	0.888
	Increased	31 /327 (9.5)	3 (6.7)	1/19 (5.3)	1 (3.4)	3 (8.1)	5 (10.6)	18/150 (12.0)	
I can understand almost all of the research ethics terms that I encounter in journal articles.	Decreased	37/329 (11.2)	5 (11.1)	4 (20.0)	2 (6.9)	0 (0.0)	3 (6.4)	23/151 (15.2)	0.182
	Increased	116/329 (35.3)	15 (33.3)	6 (30.0)	9 (31.0)	11 (29.7)	20 (42.6)	55/151 (36.4)	
To be an intelligent reader of the biomedical literature, it is necessary to know something about research ethics.	Decreased	29/327 (8.9)	2 (4.4)	2/19 (10.5)	1 (3.4)	3 (8.1)	3/46 (6.5)	18/151 (11.9)	0.655
	Increased	32/327 (9.8)	4 (8.9)	2/19 (10.5)	1 (3.4)	4 (10.8)	3/46 (6.5)	18/151 (11.9)	
*Study Design*									
I am confident I can design a study to answer a specific hypothesis.	Decreased	49/328 (14.9)	11/44 (25.0)	4 (20.0)	2 (6.9)	2 (5.4)	8 (17.0)	22/151 (14.6)	0.032*
	Increased	99/328 (30.2)	8/44 (18.2)	6 (30.0)	12 (41.4)	10 (27.0)	22 (46.8)	41/151 (27.2)	
I can understand almost all of the study design terms (e.g. sampling, case-control,	Decreased	20/328 (6.1)	3/44 (6.8)	1 (5.0)	0 (0.0)	1 (2.7)	1 (2.1)	14/151 (9.3)	< 0.001*
	Increased	128/328 (39.0)	11/44 (25.0)	9 (45.0)	21 (72.4)	15 (40.5)	25 (53.2)	47/151 (31.1)	
To be an intelligent reader of the biomedical literature, it is necessary to know something about study design	Decreased	13/325 (4.0)	2/44 (4.5)	0 (0.0)	0 (0.0)	0 (0.0)	0 (0.0)	11/150 (7.3)	0.102
	Increased	17/325 (5.2)	2/44 (4.5)	1 (5.0)	1 (3.4)	0 (0.0)	1/45 (2.2)	12/150 (8.0)	
Confidence in: *Biostatistics*	N (%)								
Interpreting the *P* value for a given result	Decreased	16/328 (4.9)	1 (2.2)	0 (0.0)	0 (0.0)	0 (0.0)	1/46 (2.2)	14/152 (9.2)	< 0.001*
	Increased	141/328 (43.0)	26 (57.8)	11 (55.0)	16/28 (57.1)	12 (32.4)	25/46 (54.3)	51/152 (33.6)	
Interpreting the implications to clinical practice for a given result from a statistical analysis	Decreased	24/330 (7.3)	1 (2.2)	2 (10.0)	1/28 (3.6)	1 (2.7)	2 (4.3)	17/153 (11.1)	0.097
	Increased	133/330 (40.3)	19 (42.2)	12 (60.0)	15/28 (53.6)	15 (40.5)	22 (46.8)	50/153 (32.7)	
Assessing if the correct statistical procedure was used to answer a research question	Decreased	45/327 (13.8)	7/44 (15.9)	9/19 (47.4)	3/28 (10.7)	3 (8.1)	5/46 (10.9)	18/153 (11.8)	< 0.001*
	Increased	94/327 (28.7)	7/44 (15.9)	4/19 (21.1)	12/28 (42.9)	12 (32.4)	10/46 (21.7)	49/153 (32.0)	
Identifying the factors that influence the adequacy of a study’s sample size	Decreased	65/330 (19.7)	11 (24.4)	13 (65.0)	4/28 (14.3)	2 (5.4)	6 (12.8)	29/153 (19.0)	< 0.001*
	Increased	84/330 (25.5)	9 (20.0)	1 (5.0)	6/28 (21.4)	13 (35.1)	19 (40.4)	36/153 (23.5)	
Evaluating diagnostic tests	Decreased	24/327 (7.3)	2/43 (4.7)	2 (10.0)	2 (6.9)	0 (0.0)	4 (8.5)	14/151 (9.3)	0.154
	Increased	152/327 (46.5)	14/43 (32.6)	10 (50.0)	18 (62.1)	21 (56.8)	25 (53.2)	64/151 (42.4)	
Analyzing the data to find association between two variables	Decreased	52/328 (15.9)	8/44 (18.2)	5 (25.0)	3 (10.3)	1 (2.7)	6 (12.8)	29/151 (19.2)	0.039*
	Increased	88/328 (26.8)	4/44 (9.1)	6 (30.0)	12 (41.4)	13 (35.1)	12 (25.5)	41/151 (27.2)	
Analyzing the data to find correlation between two variables	Decreased	57/328 (17.4)	7/44 (15.9)	6 (30.0)	2 (6.9)	1 (2.7)	9 (19.1)	32/151 (21.2)	< 0.001*
	Increased	84/328 (25.6)	4/44 (9.1)	5 (25.0)	15 (51.7)	12 (32.4)	9 (19.1)	39/151 (25.8)	
*Research Ethics*									
Applying ethical principles (e.g. confidentiality, beneficence, non-maleficence, autonomy)	Decreased	23/323 (7.1)	3 (6.7)	1/19 (5.3)	3/27 (11.1)	1 (2.7)	6 (12.8)	9/148 (6.1)	0.021*
	Increased	64/323 (19.8)	6 (13.3)	3/19 (15.8)	3/27 (11.1)	14 (37.8)	15 (31.9)	23/148 (15.5)	
*Research Ethics*									
Applying for an approval from an Institutional Review Board (IRB) / Ethics Committee	Decreased	21/324 (6.5)	7 (15.6)	3 (15.0)	1/26 (3.8)	1 (2.7)	2 (4.3)	7/149 (4.7)	0.043*
	Increased	146/324 (45.1)	14 (31.1)	5 (25.0)	10/26 (38.5)	21 (56.8)	26 (55.3)	70/149 (47.0)	
Criteria to justify authorship in research journal publications	Decreased	27/324 (8.3)	7 (15.6)	1/19 (5.3)	3/28 (10.7)	1 (2.7)	0 (0.0)	15/149 (10.1)	0.005*
	Increased	113/324 (34.9)	5 (11.1)	5/19 (26.3)	8/28 (28.6)	19 (51.4)	20/46 (43.5)	56/149 (37.6)	
Awareness of the actions that constitute publication misconduct	Decreased	47/319 (14.7)	5 (11.1)	4 (20.0)	3/28 (10.7)	2 (5.4)	3/46 (6.5)	30/143 (21.0)	0.017*
	Increased	94/319 (29.5)	9 (20.0)	3 (15.0)	6/28 (21.4)	18 (48.6)	16/46 (34.8)	42/143 (29.4)	
*Study Design*									
Knowledge of the advantages and disadvantages of each study design	Decreased	19/327 (5.8)	4/43 (9.3)	1 (5.0)	1 (3.4)	0 (0.0)	1 (2.1)	12/151 (7.9)	0.081*
	Increased	145/327 (44.3)	12/43 (27.9)	14 (70.0)	16 (55.2)	17 (45.9)	22 (46.8)	64/151 (42.4)	
Identifying ways of reducing bias when designing studies	Decreased	48/328 (14.6)	12/44 (27.3)	4 (20.0)	1 (3.4)	3 (8.1)	4 (8.5)	24/151 (15.9)	< 0.001*
	Increased	106/328 (32.3)	1/44 (2.3)	8 (40.0)	21 (72.4)	15 (40.5)	19 (40.4)	42/151 (27.8)	

^a^*JCU* James Cook University, *PUC* Pontificia Universidad Catolica de Chile, *BGU* Ben-Gurion University, *DUMC* Duke University Medical Centre, *NTNU* Norwegian University of Science and Technology, *NUS* National University of Singapore (inclusive of Yong Loo Lin School of Medicine and from Duke-NUS Graduate Medical School)

^b^Based on Chi-square test

^c^Remaining data not reported in the table had ‘no change’ in those statements

*p-value < 0.05 were statistically significant

The only factor which was significantly associated with an increased inclination towards research was increased desire to learn biostatistics (Odds Ratio (OR) 1.68; Confidence Interval (CI) 1.03–2.74) (Table 3). However, after adjusting for gender, age and institution, increased desire to learn biostatistics was no longer statistically significant. Meanwhile, absence of a role model and lack of understanding of study design terms described in journal articles were significantly associated with a decrease in inclination towards research (Table 3). After multivariate adjustment, only absence of a research role model remained statistically significant (OR 0.37; CI 0.18–0.79).

**Table 3:** Change in inclination towards research (multinomial logistic regression)

Factors	Increase inclination towards research		Decrease inclination towards research	
	Bivariate (OR)	Multivariate^a^ (OR)	Bivariate (OR)	Multivariate^a^ (OR)
Role model during medical school by final year	0.93 (0.52–1.67)	0.86 (0.46–1.61)	0.40 (0.20–0.80)*	0.37 (0.18–0.79)*
Desire to learn more about biostatistics	1.68 (1.03–2.74)*	1.64 (0.94–2.86)	0.71 (0.43–1.18)	0.76 (0.44–1.32)
Understanding of study design terms in journal articles	0.76 (0.48–1.20)	–	0.56 (0.35–0.89)*	

^a^adjusted for gender, age and institution

*statistically significant OR

Table 4 summarises the capability and confidence factors that were significantly associated with a change in inclination towards a research career. Students who reported an increased desire to learn more about biostatistics and an increased knowledge of manipulation of statistics were more likely to experience an increased inclination towards a research career. After multivariate adjustment, only an increased desire to learn more about biostatistics remained significant (OR 2.58; 1.38–4.84). Meanwhile, the factors associated with decreased inclination towards research career were an understanding of data analysis, research ethics, knowledge of study design and potential biases (Table 4). In multivariate analysis, the ability to analyse data was significantly associated with a decrease in inclination towards research career (OR 0.49; 0.31–0.78).

**Table 4:** Odds ratio (OR) using multinomial logistic regression of change in inclination towards biomedical career

Factors	Increase inclination towards research		Decrease inclination towards research	
	Bivariate (OR)	Multivariate^a^ (OR)	Bivariate (OR)	Multivariate^a^ (OR)
Had an experience during medical school that put off respondent from research by final year	0.52 (0.25–1.11)		1.43 (0.82–2.46)	
Read biomedical journals at least once every 6 months during medical school by final year	1.77 (0.99–3.17)		0.66 (0.40–1.08)	
Presence of role model during medical school by final year	1.71 (0.92–3.16)		0.73 (0.39–1.35)	
Increased desire to learn more about biostatistics	2.02 (1.18–3.46)*	2.58 (1.38–4.84)*	0.86 (0.54–1.36)	0.87 (0.50–1.51)
Increased knowledge of ease of manipulation of statistics	1.69 (1.01–2.83)*		0.86 (0.55–1.35)	
Increased confidence in analyzing the data to find association between two variables	1.18 (0.75–1.86)	1.10 (0.66–1.83)	0.47 (0.31–0.70)*	0.49 (0.31–0.78)*
Increased confidence in analyzing the data to find correlation between two variables	1.57 (0.99–2.48)		0.61 (0.41–0.90)*	
Increased confidence in evaluating diagnostic tests	1.57 (0.96–2.57)		0.76 (0.51–1.12)	
Increased understanding of research ethics terms in journal articles.	0.96 (0.61–1.51)		0.62 (0.42–0.92)*	
Increased confidence in applying for an approval from an Institutional Review Board/ethics committee	1.37 (0.84–2.25)		0.74 (0.49–1.12)	
Increased understanding of study design terms in journal articles.	1.04 (0.63–1.70)		0.60 (0.39–0.92)*	

^a^adjusted for gender, age and institution

*statistically significant OR

## Discussion

The initial investigation of inclination of medical students at entry into medical school from different parts of the world showed 35% had an inclination towards research and 25% towards a research career [4]. At follow-up, overall, there was a slight decline in inclination; 31% of students were inclined towards research and 23% inclined towards a research career. The change in inclination towards research may possibly be due to the school’s curricula, and the format of teaching in each of the medical schools.

Longitudinal studies such as the present study will assist in the assessment of current medical programmes for supporting students to undertake and be better motivated towards research, as well as improvements that could be made to curricula. It is alarming to know that year by year the numbers of dedicated academic posts are declining [6]. Thus, following our students as they complete their medical school and enter into residency allows a better understanding of their interests and the impact of the medical school curricula on research training.

While our findings suggest that medical students did not have any statistically significant changes in their interest towards research and pursuing a research career, there were a few schools with a larger proportion of students who increased their inclination towards various aspects of research. BGU in Israel provided the best learning experience to increase students’ interest and confidence in research, while Duke University in USA and NUS in Singapore both improved students’ confidence in publication ethics.

The study also showed that presence of a good research role model and knowledge in biostatistics and study design/research methodologies were key influences in changes of inclination towards research and research careers. It is known that when the ease of access to readily available information is present, the likelihood of students wanting to pursue a research opportunity also increases [5]. Good and reliable resources that are readily available are important in encouraging medical students to incorporate research into their activities.

There have been few other similar studies on factors that influence the pursuit of research or research careers preferences by medical students [11–13] and studies on numbers of medical students that have research career plans [14, 15]. Our study suggests a worrying trend of consistently low interest in research among medical students. Similarly, [3] Guelich et al. showed a declining trend of research intentions over the span of a decade between 1987 and 1997. Our follow-up study identifies some of the factors and barriers associated with interest in research, and these should be considered in reviewing medical curricula to allow for greater emphasis on the training of clinician scientists. In addition, a previous study suggested medical schools should improve their school curricula by portraying medical research as a rewarding and enjoyable career [3], while a more recent study suggested the curriculum could incorporate compulsory group-based research activities that are well supported as a way to motivate students to undertake research and potentially careers in research [7].

In order to stem the declining interest of future clinicians towards research and research careers, current medical curricula should address barriers to student research, and adopt motivational strategies for research activity. Barriers such as inadequate funding for research, differential in pay between community and academic centres, and lack of proper initial mentorship during the transition period from resident to independent investigator have been noted as early as 2002 [16]. Barriers such as time and the perception that students would not receive appropriate acknowledgement for work contributed to a research project should also be addressed [13]. Early exposure to research may also help demystify the process of conducting research and render it more likely that research activities will be pursued during residency and potentially as a career [5].

## Limitations

There are several limitations to this study. All capabilities and confidence towards research were self-reported. As expected, there could be bias in perceived knowledge of statistics and medical research among the medical students. Also, as this was a prospective cohort study, following students up from different medical schools across varying countries was challenging, with the loss to follow up being 57%. Thus, the study is subjected to responder biases. Reasons for this significant loss were the long follow-up period, turnover of faculty staff, students being busy in their final year, and some students being posted to different training sites during their final year thus making it difficult to contact them for the survey. Other limitations include variations in medical school duration and follow-up across different countries. However, the follow-up process was standardised as much as possible by administering the questionnaire the same way it was at entry into medical school, and adjusting for institutional effects in multivariate models. Despite the limitations of missing data and a low follow up response rate, some cohorts demonstrated an increased inclination towards research of 22%, with 35% were less inclined. These figures may be what can realistically be expected from a cohort of medical students in terms of their interest in pursuing research careers. Due to the varying numbers of students and the small number of students in some schools, this may limit the comparisons made among the schools. However, providing results by school as well as an aggregate is a starting point for further investigations as to why differences exists in the interests towards pursuing research degrees. Examination of differences in medical school curricula may allow more specific changes to be made to encourage and inspire students to pursue research careers. We also did not examined the effect of having a previous degree on their interest in pursuing biomedical research as there were only a small number of students (26%) that had a previous degree before pursuing a medical degree. We acknowledged that this could be a potential confounding factor in our analysis, hence the conclusion of this study to be made with caution.

## Conclusions

With caution of the limitations of this study, we conclude that most medical students do not change their inclination towards research after completing their undergraduate medical training. This finding also suggests that for medical students to improve their inclination towards research, the medical curricula should be changed to incorporate more research activities, and greater attention should be given to building students’ knowledge of the research process and methods of statistical analysis [17]. In addition, good research role models should be made available to interested students [18], which may require an incentive system to encourage researchers to be role models. As training clinicians who can play his/her part in medical research is important for the future of medicine, medical schools could also consider selecting medical students based on their past research activities and their prior interest in research at time of application.

## Abbreviations

BGU: Ben-Gurion University (BGU) Israel
JCU: James Cook University
NTNU: Norwegian University of Science and Technology (NTNU)
NUS: National University of Singapore (NUS)
OR: Odds Ratio
PhD: Doctorate of Philosophy
PUC: Pontificia Universidad Catolica de Chile (PUC)
SD: Standard Deviation
USA: United States of America
